# Genetic and therapeutic landscapes in cohort of pancreatic adenocarcinomas: next-generation sequencing and machine learning for full tumor exome analysis

**DOI:** 10.18632/oncotarget.28512

**Published:** 2024-02-05

**Authors:** P.A. Shatalov, N.A. Falaleeva, E.A. Bykova, D.O. Korostin, V.A. Belova, A.A. Zabolotneva, A.P. Shinkarkina, A. Yu Gorbachev, M.B. Potievskiy, V.S. Surkova, Zh V. Khailova, N.A. Kulemin, Denis Baranovskii, A.A. Kostin, A.D. Kaprin, P.V. Shegai

**Affiliations:** ^1^National Medical Research Radiological Centre of the Ministry of Health of the Russian Federation, Obninsk 249036, Russia; ^2^Center for Precision Genome Editing and Genetic Technologies for Biomedicine, Pirogov Russian National Research Medical University, Moscow 117997, Russia; ^3^FSBI “Lopukhin Federal Research and Clinical Center of Physical-Chemical Medicine” FMBA, Moscow 119435, Russia; ^4^Peoples Friendship University of Russia (RUDN University), Moscow 117198, Russia

**Keywords:** pancreatic cancer, tumor mutation burden, somatic mutations, artificial intelligence, machine learning

## Abstract

About 7% of all cancer deaths are caused by pancreatic cancer (PCa). PCa is known for its lowest survival rates among all oncological diseases and heterogenic molecular profile. Enormous amount of genetic changes, including somatic mutations, exceeds the limits of routine clinical genetic laboratory tests and further stagnates the development of personalized treatments. We aimed to build a mutational landscape of PCa in the Russian population based on full exome next-generation sequencing (NGS) of the limited group of patients. Applying a machine learning model on full exome individual data we received personalized recommendations for targeted treatment options for each clinical case and summarized them in the unique therapeutic landscape.

## INTRODUCTION

According to the American Cancer Society’s (ACS) pancreatic cancer (PCa) accounts for about 7% of all cancer deaths and holds a 5-year survival rate lower than 50% [1]. PCa is a specific diagnostic and therapeutic problem among all oncological diseases. Tumor is symptomless at the early stages. According to an analysis of the main parameters of oncological healthcare in Russia in 2021, PCa was detected at stage IV in 59.5% of patients [2]. Survival rates remain dramatically low: first-year mortality in patients with metastatic PCa is 67.3%, and 5-year survival rate is 3% [2, 3]. While chemotherapy is the main treatment strategy for metastatic PCa, all the common regimes failed to improve the progression-free survival rate significantly [4, 5]. Insufficient progress in novel effective drug development is probably associated with a misunderstanding of genomic and molecular mechanisms of tumor chemo-resistance and progression [6]. Heterogeneous tumor geno- and phenotype and variative cellular microenvironment of PCa determine cell pathways for drug evasion [5, 7, 8]. Novel promising therapeutic approaches for PCa are based on the administration of targeted treatment and immunotherapy based on personalized screening of tumor mutational profile. The development of next generation sequencing (NGS) during the last decade exploded the scientific and clinical interest in genomic research. In recent years it has emerged as a powerful platform for future targeted treatments based on personalized approaches [9].

On average, up to 63 mutations could be detected in each sample of pancreatic adenocarcinoma. Those include 12 well-known mutations associated with main signal pathways. According to the cancer genome atlas, mutations commonly appear in the KRAS pathway (more than 90% of invasive PCa). However, other driver mutations also could be detected, including Ink4a, BRCA2, LKB1, P16/CDKN2A, p53, SMAD4, MLH1, PRSS1; BRAF, MAPK, PI3K, Akt; VEGF, and IGF1R.

The extraordinary potential of NGS to accurately identify genomic variations and repetitive molecular-genetic abnormalities paved the way for novel diagnostic tools in clinical oncology. Employing NGS allows clinical trials of personalized approaches based on genomic biomarkers or other mutation-specific agents [6].

Somatic structural chromosome rearrangement is a general mutation class associated with gene damage (e.g., deletion or rearrangement), gene activation (e.g., copy number increase or amplification), and the formation of new gene products (gene fusion). Most of them stimulate cancerogenesis and thus may be considered therapeutic targets. Complex and widespread patterns of chromosome rearrangements were observed in PCa as well. Furthermore, significant heterogeneity of tumor mutational landscapes in various demographic groups and populations obstructs wide clinical translation of PCa personalized treatment [5, 10]. A growing body of literature recognises the importance of mutational and treatment landscapes for PCa in different populations [11]. Despite the considerable clinical attention only a few studies have recently attempted to investigate metastatic PCa in a localized population applying NGS in any systematic way [12].

In our study, we examined 40 tumor and blood samples obtained from patients with PCa at different stages. We aimed to collect their complete mutational profiles using full exome NGS analysis. We also proposed personalized targeted treatment options, applying artificial intelligence on individual exome data. Finally, we built genetic and therapeutic landscapes for PCa in our population.

## RESULTS

According to the molecular-genetic examination, it was revealed that 1 patient (2.5%) had an unstable microsatellite status of the tumor (MSI-H).

**Figure 1:**
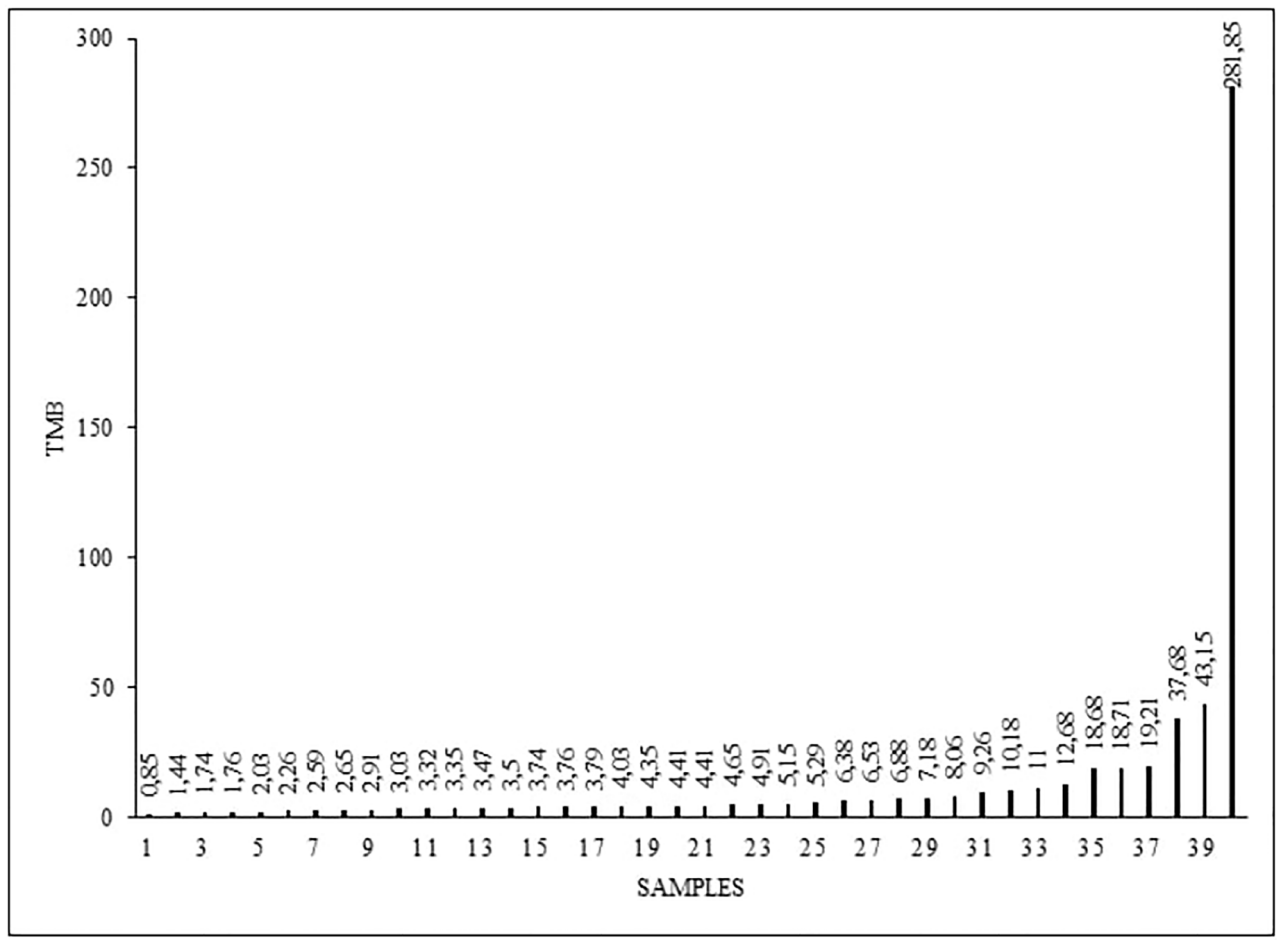
Tumor mutation burden (TMB) levels (mut/mb) in 40 analyzed samples.

We detected different tumor mutation burden (TMB) levels in the analyzed samples, in range from 0,85 to 281,85, with the median of 4,41 TMB (Figure 1). High TMB (more than 10 mut/Mb) was detected in 9 patients.

**Figure 2:**
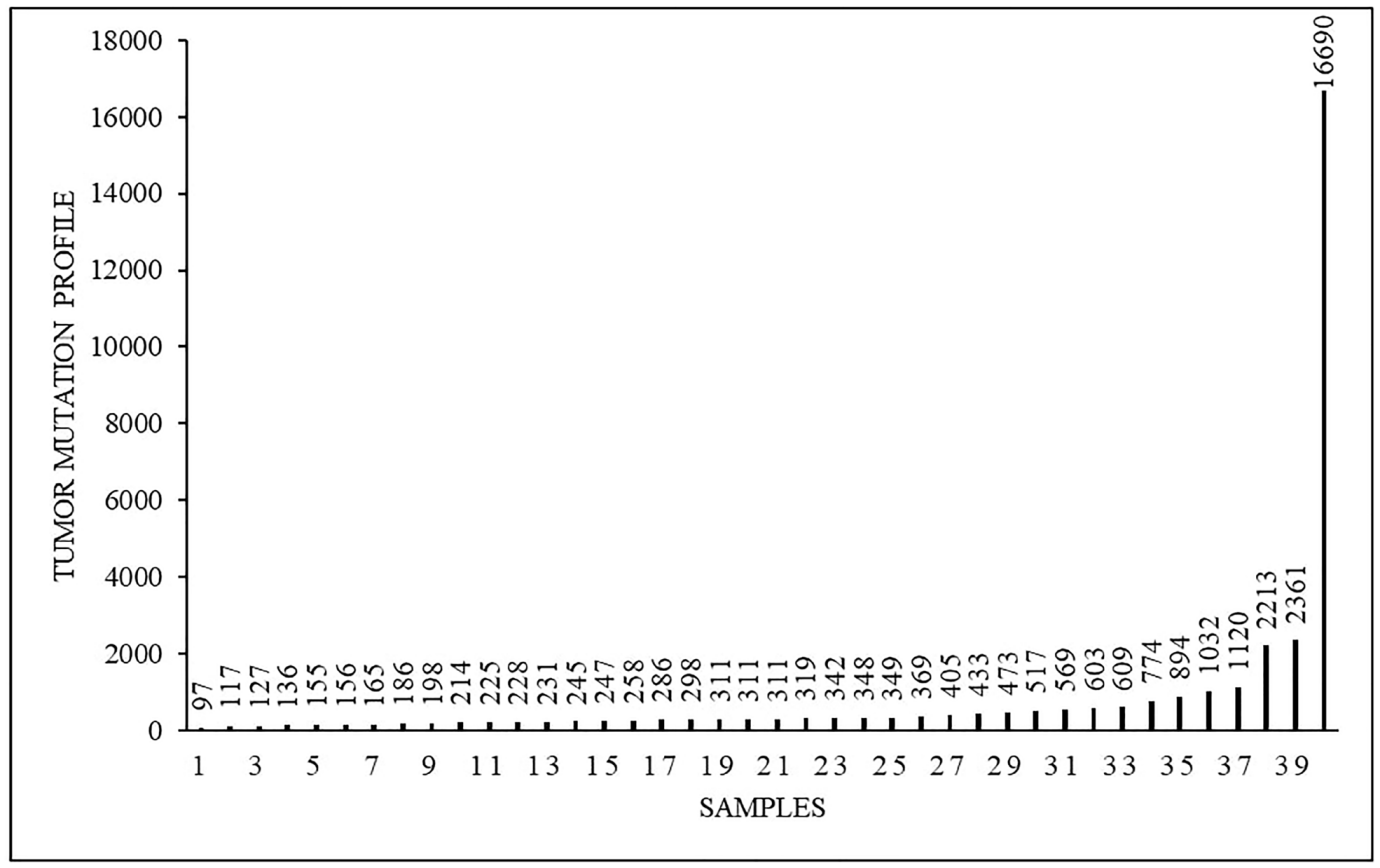
Tumor mutational profile (mut) in 40 analyzed samples.

Tumor mutational profile was heterogeneous and varied from 97 to 16690 somatic mutations with an average amount of 873 (Figure 2). Totally we identified a pool of 22091 somatic tumor-specific mutations in the whole group of 40 patients. For further analysis we selected 400 genes, as their mutations were non-unique in our population, while we observed them in more than just one patient.

At the same time, we detected somatic mutations of pathogenicity class 1 (driver mutations) in 31 patients (77.5%) according to the guidelines for the interpretation of clinically significant somatic mutations detected by NGS in solid tumors. All patients had mutations in the *KRAS* gene (Table 1).

**Table 1:** Somatic mutations of the pathogenicity class 1

Gene	Mutations, genomic changes/protein changes
*KRAS*	12:g.25398281C>T/p.Gly13Asp;
	12:g.25398282C>A/p.Gly13Cys;
	12:g.25398284C>A/p.Gly12Val;
	12:g.25398284C>T/p.Gly12Asp;
	12:g.25398285C>G/p.Gly12Arg;
	12:g.25398285C>A/p.Gly12Cys

*KRAS* mutation is known as a poor prognosis factor, as patients with this mutation have significantly shorter overall survival regardless of chemotherapy [13]. According to the randomized multicenter trials PRODIGE 4/ACCORD 11 (2011), the median overall survival (OS) was 11.1 months in the group of metastatic patients who underwent chemotherapy in FOLFIRINOX regime [14]. The median OS was 11 months in the group of patients with a mutation in the *KRAS* gene studied in our research. In our study, 17 patients of 31 received adaptive immunotherapy with allogeneic activated *in vitro* lymphocytes in parallel with standard treatment options. The median OS in this group of patients was 12 months.

5 patients (12.5%) had somatic mutations of pathogenicity class 2 in the *TP53* gene (tumor suppressor gene) (17:g.7577538C>T/p.Arg248Gln; 17:g.7578406C>T/p.Arg175His). Co-occurrence of tumor pathogenic mutations in *KRAS* and *TP53* was observed in 4 patients (10%).

Pathogenicity class 2 mutation in the *KIT* gene (the gene encoding the transmembrane protein receptor tyrosine kinase or CD117) (4:g.55593464A>C/p.Met541Leu) was found in 1 (2.5%) patient.

**Figure 3:**
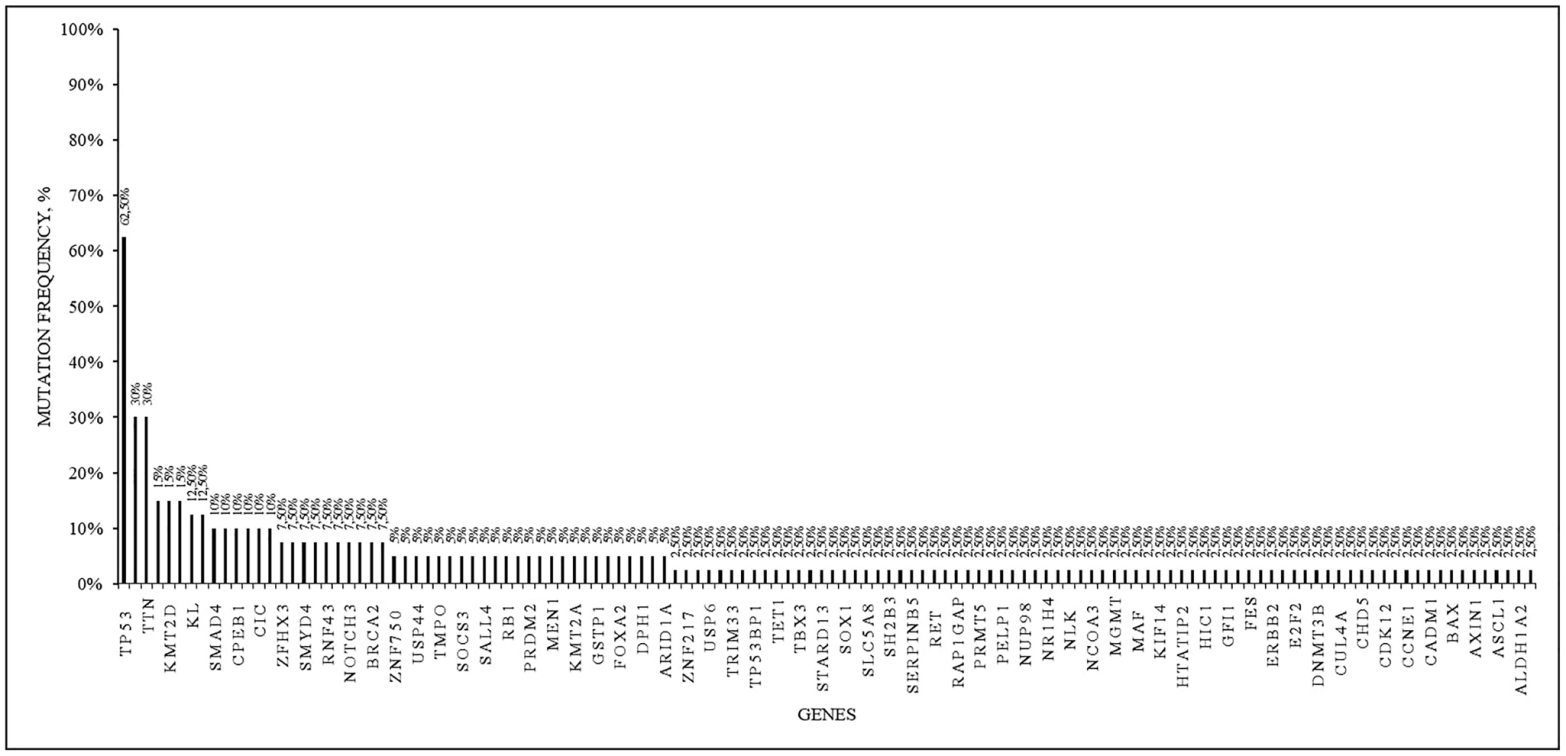
Somatic mutations of the pathogenicity class 3 in 40 analyzed samples. The frequency among the samples is presented.

We detected 26 mutations of pathogenicity class 3 in the *TP53* gene in 25 patients (62.5 %); 12 patients (30%) had 42 mutations of pathogenicity class 3 in the *TTN* gene, 6 patients (15%) had mutations in the *KMT2D* gene, 5 patients (12.5%) had somatic mutations of pathogenicity class 3 in the *KL* gene. In 4 patients (10%) mutations of pathogenicity class 3 in the *CPEB1, SMAD4* and *CIC* genes were found (Figure 3, Table 2).

**Table 2:** The most common somatic mutations of the pathogenicity class 3

Gene	N mutated patients, abs (%)	Mutations, genomic changes/protein changes
*TP53*	25 (62,5%)	17:g.7574002CG>C/p.Arg342GlufsTer3
		17:g.7577022G>A/p.Arg306Ter,
		17:g.7577094G>A/p.Arg282Trp,
		17:g.7577097C>G/p.Asp281His,
		17:g.7577142C>T/p.Gly266Arg,
		17:g.7577497A>C/NA,
		17:g.7577498C>T/NA,
		17:g.7577526AG>A/p.Leu252SerfsTer93,
		17:g.7577547C>A/p.Gly245Val,
		17:g.7577566T>TA/p.Asn239Ter,
		17:g.7577581A>T/p.Tyr234Asn,
		17:g.7577593TAC>T/p.Cys229TyrfsTer10,
		17:g.7578190T>C/p.Tyr220Cys,
		17:g.7578212G>A/p.Arg213Ter,
		17:g.7578246CA>C/p.Leu201CysfsTer46,
		17:g.7578271T>C/p.His193Arg,
		17:g.7578394T>C/p.His179Arg,
		17:g.7578451ATGGCGC>A/p.Arg158_Met160delinsLeu,
		17:g.7578524G>A/p.Gln136Ter,
		17:g.7578530A>G/p.Phe134Leu,
		17:g.757930A>T/NA,
		17:g.7579358C>G/p.Arg110Pro,
		17:g.7579472G>C/p.Pro72Arg,
		17:g.7579590A>AAC/p.Ser33ValfsTer12.
*TTN*	12 (30%)	2:g.179393691G>A/p.Thr35596Ile,
		2:g.179396082G>A/p.Thr35087Met,
		2:g.179396766C>T/p.Arg34859Gln,
		2:g.179397561C>T/p.Arg34594His,
		2:g.179406191C>T/p.Arg32538His,
		2:g.179421694A>G/p.Ile29396Thr,
		2:g.179424952G>A/p.Pro28636Leu,
		2:g.179427536T>C/p.Ile27775Val,
		2:g.179430275T>C/p.Ser26862Gly,
		2:g.179430997G>A/p.Thr26621Met,
		2:g.179431076C>G/p.Asp26595His,
		2:g.179432185A>G/p.Ile26225Thr,
		2:g.179436020G>A/p.Arg24947Cys,
		2:g.179437195A>T/p.Ile24555Asn,
		2:g.179444939C>T/p.Val22359Ile,
		2:g.179457147G>A/p.Pro19862Leu,
		2:g.179457639G>A/p.Ser19736Leu,
		2:g.179458591C>T/p.Arg19479His,
		2:g.179464527T>C/p.Asn18701Asp,
		2:g.179529425G>A/p.Thr12053Met,
		2:g.179545859C>T/p.Arg11096His,
		2:g.179554305C>T/p.Gly10622Arg,
		2:g.179558366T>C/p.Ile10522Val,
		2:g.179569387T>A/p.Thr9938Ser,
		2:g.179569986C>A/p.Arg9840Leu,
		2:g.179579093T>C/p.Asn8803Ser,
		2:g.179582537G>T/p.Ala8355Glu,
		2:g.179583496T>G/p.Glu8144Ala,
		2:g.179586604C>G/p.Asp7596His,
		2:g.179587130C>G/p.Asp7462His,
		2:g.179600475C>T/p.Ala4900Thr,
		2:g.179617869T>C/p.Ile3765Val,
		2:g.179631240G>C/p.Gln3191Glu,
		2:g.179638721C>T/p.Gly2392Ser,
		2:g.179658175C>T/p.Val498Ile,
		2:g.179659912G>A/p.Arg328Cys.
*KMT2D*	6 (15%)	12:g.49427265TTGC>T/p.Gln3745del,
		12:g.49434913C>T/p.Ala2214Thr,
		12:g.49441799AAGG>A/p.Leu1395del,
		12:g.49448520C>T/p.Arg64Gln.
*LRP1B*	6 (15%)	2:g.141116420C>T/p.Gly3743Ser,
		2:g.141116447G>T/p.Gln3734Lys,
		2:g.141458166T>A/p.Asn2151Ile,
		2:g.141526839C>T/p.Asp1901Asn,
		2:g.141707933C>A/p.Val1003Phe,
		2:g.142012098G>T/p.Ser152Arg,
		2:g.142012126C>G/p.Gly143Ala,
		2:g.142567910T>C/p.Gln48Arg.
*KL*	5 (12,5%)	13:g.33591042G>A/p.Arg155Gln,
		13:g.33628138T>G/p.Phe352Val,
		13:g.33628193G>C/p.Cys370Ser,
		13:g.33634897G>T/p.Asp561Tyr.
*CPEB*	4 (10%)	15:g.83222225G>A/p.Pro352Leu,
		15:g.83222235G>A/p.Pro349Ser,
		15:g.83296073C>T/p.Ala21Thr.
*SMAD4*	4 (10%)	18:g.48575159C>T/p.Ala118Val,
		18:g.48591919G>A/p.Arg361His,
		18:g.48593417G>T/p.Glu390Ter.
*CIC*	4 (10%)	19:g.42795382G>A/p.Gly821Glu,
		19:g.42798376G>C/p.Arg1416Thr,
		19:g.42799299T>C/p.Ser1595Pro.

**Figure 4:**
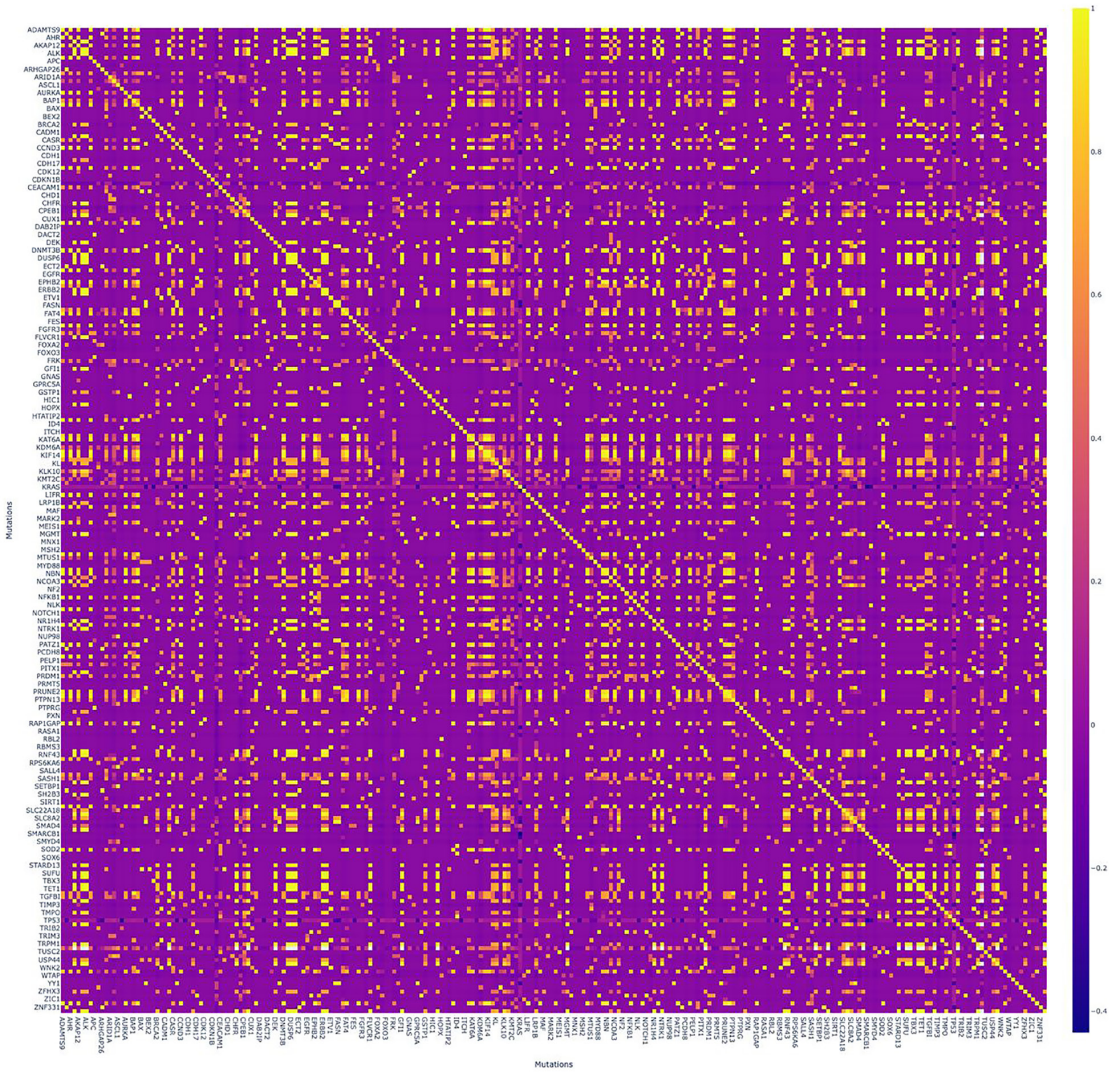
Correlation matrix with numerous significant positive correlations. Pearson criteria was applied for each pair of genes. Colored scale (on the right) - “Turbo”: positive correlations are bright/yellow, negative are dark/blue.

The observed mutation and therapeutic profile was summarized in the landscape including heatmap, combined with bar-plots illustrating the total number of mutations (Figure 4). One patient was excluded from the heatmap due to extremely high mutation burden and a total count over more than 16 000 somatic mutations.

**Figure 5:**
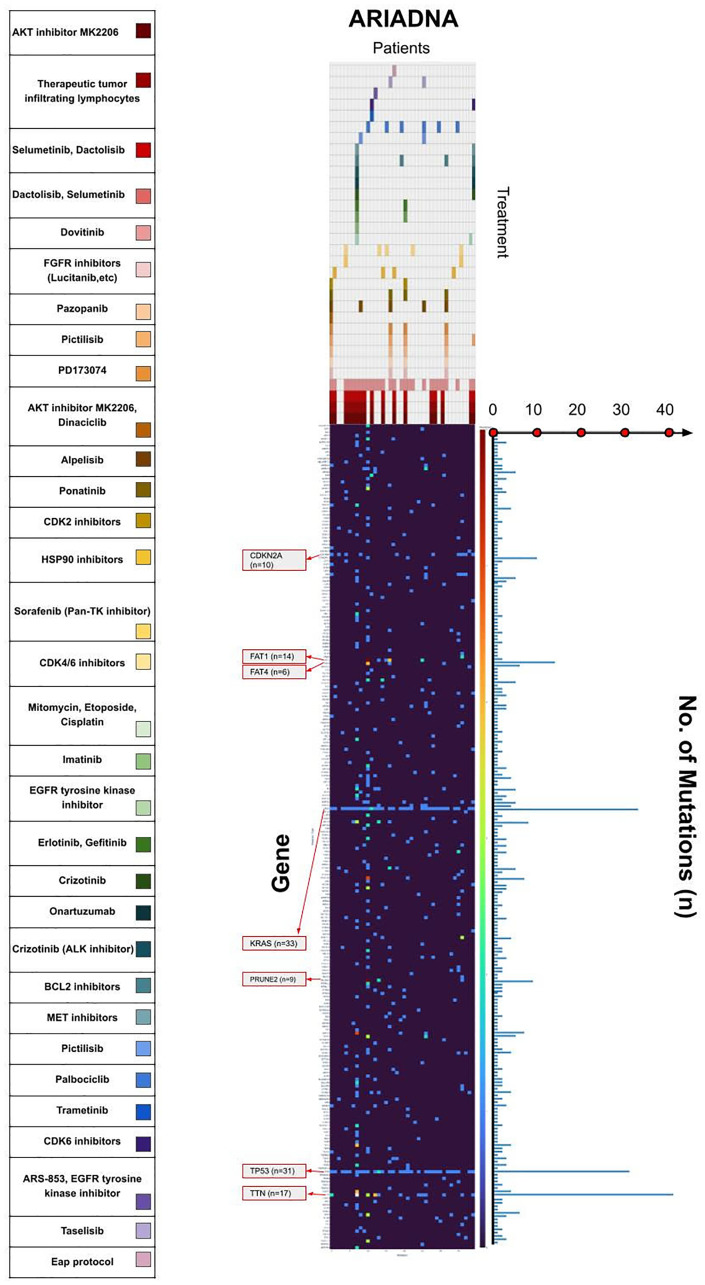
Mutational and therapeutic landscape of PCa in the Russian population. Heatmap in the middle illustrates the quantity of mutations for each gene in each patient in our group- Color brightness increases from the dark blue via bright yellow to red representing the number of mutations. Therapeutic landscape on the top illustrates the therapy recommended for each patient by artificial intelligence software based on machine learning (ARIADNA, Russia). Each color corresponds to one recommended drug according to the large colored scale on the left side.

We also built a correlation matrix applying Pearson criteria for each pair of genes, appearing with numerous significant positive correlations (Figure 5).

However, we did not detect other genetic abnormalities common in PCa and presented in the literature, such as mutations of genes involved in the repair of DNA damage (*BRCA1/2, ATM, BAP1, RAD50, FANCE, PALB2*), chromatin remodeling (*ARID1A, PBRM1, ARID2, KMT2D, KMT2C, SMARCA4, SETD2*) and cell cycle control pathways (*CDKN2A, CCND1, CCNE1*), as well as in *Ink4a*, *LKB1, MLH1, PRSS1*, BRAF, MAPK, PI3K, Akt, VEGF and IGF1R genes.

## DISCUSSION

NGS represents the greatest promise for precision oncology, being capable of detecting rare oncogenic mutations with therapeutic potential. Our study was designed to build a specific mutational and therapeutic landscapes of PCa among the Russian population. We obtained the first mutation landscape for 40 Russian patients with PCa and described prevalence in *KRAS, TP53*, and *TTN* genes with 33, 31 and 17 mutations in total, respectively. Total number of mutations per patient as well as patients age were found completely independent and heterogeneous. Similar landscape was confirmed by the retrospective analysis of whole-exome PCa DNA sequencing in the Greek population, excluding *KRAS* mutation rate [15]. Intriguingly, low *KRAS* mutation rate in the Greek population, compared to the Russian population in our study, is the most striking observation to emerge. *KRAS* mutation was found to be the most frequent in our analyzed group being detected in each patient. Previously, the incidence of *KRAS* was extremely poorly understood among the patients with PCa in Russia, while overall world data reported *KRAS* pathway mutations for more than 90% cases of metastatic PCa and 65.5% of all the patients with the disease [10, 16]. The differences may be also associated with ecological, ethnic and geographic features, being a good subject for further investigations. Patients with *KRAS* mutation are expected to have poorer survivability and higher mortality. The coexistence of *KRAS* and *TP53* mutations may play a crucial role in PCa pathogenesis and seem to have a negative influence on the treatment outcomes in patients, receiving cytotoxic drugs or anti-EGFR/Akt/mTOR target therapy. We found a slightly positive correlation coefficient (CC = 0.41) for the pair *KRAS/TP53* in our study. The last finding therefore needs to be interpreted with caution due to the small group size. *TP53* is a known tumor suppressor gene that activates a response to cellular stress and DNA damage when the cell cycle process is stopped. The unfavorable prognostic value of *TP53* mutations is well known. Mutations in *TP53* are usually found in approximately 60-70% of PCa, corresponding to 62.5% of patients in our study with a maximum of 4 mutations per patient [1, 17]. Except for each other, both *KRAS* and *TP53* had no significant correlations in mutations count with any other genes. However, correlation matrix demonstrates a high number of positive correlations with *TTN* gene, including *AIM2, ALK, CHFR, DEK, TET1, TGFBR2* etc. (CC > 0.97) and *BRCA2* (CC = 0.81). The *TTN* gene contains the largest number of exons of all known genes and encodes the titin protein, the largest of the single peptides. Titin plays a key role in the assembly of sarcomeres and the transmission of muscle contraction. *TTN* mutation correlates with a better response to immune checkpoint inhibitor therapy in solid tumors, but underlying mechanisms are still unclear [18]. *TTN*-mutated cancer has been shown to have a higher TMB [19]. In our cohort we identified 12 patients with *TTN*-mutated PCa (30%) with the median TMB of 6.235 mut/Mb.

Cytoplasmic polyadenylation element-binding protein 1 (*CPEB1*), a sequence-specific RNA-binding protein that regulates polyadenylation and mRNA translation, is associated with cancer progression and metastasis. We found mutated *CPEB1* to have a strong positive correlation with affected *KMT2A* (CC = 0,81). However, the involvement of *CPEB1* in the development of PCa remains unclear [20].

The *SMAD4* gene is frequently mutated in PCa, correlates with changes in altered histopathological transitions, metastatic disease, and poor prognosis and is associated with a higher mortality rate in patients receiving anti-EGFR/Akt/mTOR therapy [15]. Loss of *SMAD4* does not change the growth rate of the primary tumor, but plays a direct role in promoting metastasis. Two out of four patients with *SMAD4* mutated PCa were diagnosed already at stage IV of the disease [21]. We found *CPEB1* and *SMAD4* mutations in 10% of patients. Importantly, our data have shown that *SMAD4* has almost zero correlation with other mutations and has to attract suitable alertness.

The *KMT2* family of histone-modifying proteins (lysine methyltransferases) plays an important role in the regulation of developmental pathways. Mutations in the *KMT2C/D* encoding genes contribute to carcinogenesis and are closely associated with many types of blood cancers and solid tumors [22]. In our study 15% of patients had mutations in the *KMT2D* gene.

In accordance with the mutational landscape and correlation matrix we could conceivably hypothesize that most commonly affected genes (*KRAS, TP53, TTN*) should be examined almost always independently, while a link may exist between the majority of others. Further clinical investigations enrolling extended groups of patients could help to extend the database, concretize our findings and finally provide a bundle of correlated genes minimizing the costs for clinical NGS analysis.

The results of NGS among the Russian population of patients with PCa differ from the other populations. A natural progression of our research is to discover and analyze the differences between the literature describing mutational profiles and our own findings. However, the scope of our study was limited by the localized and relatively small cohort of patients. Currently we refrain from generalizing our findings; however, we are convinced that a greater focus on NGS would help us to establish a greater degree of accuracy on the mutational landscape.

At the same time, it will be possible to determine the clinical significance of the identified recurring mutations with uncertain malignant potential and, possibly, to make an attempt to develop new targeted drugs taking into account the detected correlations. Considerably, NGS-powered personalized medicine may improve future survival rates for no-hope groups of patients with certainly limited therapeutic alternatives.

By applying artificial intelligence and machine learning, we requested personalized recommendations for targeted therapy for each patient in our cohort. Based on an individual mutational map, ARIADNA found the majority of patients sensitive to dactolisib and selumetinib therapy (recommended for 27 patients). Corresponding to the recent publications of clinical trials, selumetinib was found to be well-tolerated with a manageable safety profile for patients with *KRAS*-mutated and metastatic PCa and was able to support a limited disease stabilization [23, 24]. We also identified most of the patients as relevant candidates for AKT inhibitor-targeted treatment and cell therapy with tumor-infiltrating lymphocytes. For patients with high mutational load EGFR tyrosine kinase inhibitor and palbociclib were recommended.

In conclusion, our therapeutic landscape provides visual understanding of the distribution of targeted therapy in the population of patients with PCa. Despite the limited cohort and query for further investigations to support our observations, we found it helpful to identify the most demanded drugs with proven clinical efficacy. We are convinced that therapeutic landscapes supported by machine learning approaches could be an important tool with economic significance applicable in planning and forecasting healthcare needs.

## MATERIALS AND METHODS

### Tumor samples

In accordance with primary inclusion criteria (histologically verified and TNM-staged PCa and age over 18 years) we enrolled 60 patients with verified PCa. All patients were treated at the National Medical Radiological Research Centre of the Ministry of Health of the Russian Federation (NMRRC) within the period from 2016 to 2022. Written informed consent has been obtained from each subject. The study has been approved by the ethics committee of NMRRC (protocol No. 885). The tumor samples were obtained during planned surgical treatment or core-biopsy and presented in paraffin blocks.

The studied group included 26 males and 14 females with the age ranging from 40 to 79 years, the median age was 63. In 19 patients (47.5%), the tumor was found in the head of the pancreas, in 8 patients (20%) in the tail, in 5 patients (12.5%) the tumor affected the body and tail of the pancreas, and in 8 patients (20%) only the pancreas body.

According to morphological and immunohistochemical investigations, ductal adenocarcinoma was diagnosed in 38 patients: 9 (24%) had a highly differentiated tumor, 8 (21%) had a moderately differentiated tumor, 6 (16%) had a low-differentiated tumor, 2 (5%) had an undifferentiated tumor, and in 13 (34%) the differentiation was not determined. In 1 patient, the pancreatic tumor had a mixed character: most of the tumor was represented by a low-differentiated acinar adenocarcinoma (about 90%), while the smaller part was represented by moderately differentiated ductal adenocarcinoma. Biopsy material of 1 patient contained mucinous adenocarcinoma.

The disease was staged in accordance with TNM classification. We detected stage IB in 1 patient (2.5%), IIA in 2 (5%), IIB in 4 (10%), III in 8 (20%), IV in 25 (625%) patients. All patients underwent chemotherapy using different regimens (Table 3).

**Table 3:** Demographic and clinical parameters of patients in the study

**Characteristics**	**All patients**
***N* = **	
**Age (years)**	40
Minimum	40
Maximum	79
Median	62,5
**Sex**	
male (*n* =)	26 (65%)
female (*n* =)	14 (35%)
**Tumor localization**	
Head	19 (47,5%)
Corpus	8 (20%)
Cauda	8 (20%)
Corpus and Cauda	5 (12,5%)
**Stage**	
IB	1 (2,5%)
IIA	2 (5%)
IIB	4 (10%)
III	8 (20%)
IV	25 (62,5%)
**Tumor grade**	
Not defined	14 (35%)
Undifferentiated	2 (5%)
Low differentiated	6 (15%)
Moderately differentiated	8 (20%)
Highly differentiated	9 (22,5%)
Mixed: Low and moderately differentiated	1 (2,5%)

### DNA extraction and library preparation

DNA was extracted from peripheral blood lymphocytes with QIAamp DNA Blood Mini Kit and the QIAamp DNA FFPE Tissue Kit was used to extract DNA from paraffin-embedded tumor samples according to the manufacturer protocol.

Libraries were prepared from 100–400 ng of genomic DNA extracted from peripheral blood lymphocytes and formalin-fixed, paraffin-embedded (FFPE) blocks using the MGIEasy Universal DNA Library Prep Set (MGI Tech) according to the manufacturer’s protocol. DNA from paraffin-embedded tissues was fragmented in accordance with the protocol based on S1-nuclease employing, which cuts DNA at the nick sites [25]. To remove artifacts and achieve full DNA fragmentation, the samples were processed with “enzyme mix” USER (New England Biolabs), including uracil-DNA-glycosylase and endonuclease VIII. The enzymes release uracil in points of cytosine deamination with single-strand DNA braking. Concentrations of DNA and prepared libraries were evaluated with Qubit and dsDNA HS Assay Kit according to the manufacturer’s protocol. The quality of the libraries was checked on a Bioanalyzer 2100 with a High Sensitivity DNA kit (Agilent Technologies) according to the manufacturer’s protocol.

DNA libraries enrichment was performed according to the method with SureSelect Human All Exon V6/V7 (Agilent Technologies, USA), including the whole human exome (more than 22000 genes). DNA concentration evaluation was performed using fluorimeter Qubit 2.0 (Life Technologies). The quality control was performed using bioanalyzer Agilent Bioanalyzer 2100 (Agilent).

Then, the libraries were circularized and sequenced on the platform MGISEQ-2000 in regimes PE100 and PE150 according to the manufacturer’s protocol (MGI Tech) with an average coverage of 100× for blood samples and 200× for tumor samples.

From the whole pool of all the identified somatic tumor-specific mutations we extracted specifically non-unique ones and used them for further analysis.

### Bioinformatics data processing

FastQ files were generated using ZebracallV2 software as described previously [26]. The data quality was evaluated using FastQC software package [27], and low-quality reads were filtered and cut. The results were aggregated with the MultiQC program.

At the next step we aligned the filtrated and cut reads to the reference genome to receive a BAM file from fastq. We used the software package Burrows-Wheeler Aligner (BWA-MEM) with default settings [28]. To evaluate the reads quality SAMtools software package, bamstats module package was used [29, 30].

Variation calling was performed for .BAM file, using software packages: SAMtools, Strelka2, Sentieon, GATK [29, 31–33]. Copy number variation (CNV) calling was performed with software package CNV-Kit with learning on 10 reference exome datasets acquired on MGISEQ-2000.

Mutational Signature analysis was performed using the software package deconstructSigs [34] in order to detect mutational signatures for each tumor. 30 mutational signatures from Cosmic database were used (Mutation Signatures v3).

To calculate microsatellite instability, we used the MSI statistical identifier. Based on somatic mutation variants, the classifier is able to differentiate MSH (high microsatellite instability) and MSS (stable) tumors. The classifier was learned with 999 exome datasets from the Cancer Genome Atlas (TCGA) with known MSI status.

Visual Studio Code (v.1.78.1) was used for data analysis, preparation of heatmap, correlation matrix and mutational landscape.

We applied artificial intelligence software based on machine learning (ARIADNA, Russia) for our NGS-data. ARIADNA uses structured open information as literature and databases containing data on “*gene-drug”* associations and ranks genomic variants according to the degree of clinical significance in order to predict the most effective therapeutic approach. We collected the information on targeted treatment proposed by the artificial intelligence and built mutational and therapeutic landscapes of PCa.

## Abbreviations

ECOG: Eastern Cooperative Oncology Group
NGS: Next Generation Sequencing
PCa: pancreatic cancer
